# Spinal Cord Molecular and Cellular Changes Induced by Adenoviral Vector- and Cell-Mediated Triple Gene Therapy after Severe Contusion

**DOI:** 10.3389/fphar.2017.00813

**Published:** 2017-11-13

**Authors:** Andrei A. Izmailov, Tatyana V. Povysheva, Farid V. Bashirov, Mikhail E. Sokolov, Filip O. Fadeev, Ravil R. Garifulin, Boris S. Naroditsky, Denis Y. Logunov, Ilnur I. Salafutdinov, Yuri A. Chelyshev, Rustem R. Islamov, Igor A. Lavrov

**Affiliations:** ^1^Department of Biology, Kazan State Medical University, Kazan, Russia; ^2^Gamaleya Research Institute of Epidemiology and Microbiology, Moscow, Russia; ^3^Institute of Fundamental Medicine and Biology, Kazan Federal (Volga Region) University, Kazan, Russia; ^4^Kazan Scientific Center, Kazan Institute of Biochemistry and Biophysics, Russian Academy of Sciences, Kazan, Russia; ^5^Department of Neurologic Surgery, Mayo Clinic, Rochester, MN, United States; ^6^Department of Physiology and Biomedical Engineering, Mayo Clinic, Rochester, MN, United States

**Keywords:** spinal cord injury, glial cells, gene therapy, human umbilical cord blood mononuclear cell, vascular endothelial growth factor, glial cell-derived neurotrophic factor, angiogenin, neural cell adhesion molecule

## Abstract

The gene therapy has been successful in treatment of spinal cord injury (SCI) in several animal models, although it still remains unavailable for clinical practice. Surprisingly, regardless the fact that multiple reports showed motor recovery with gene therapy, little is known about molecular and cellular changes in the post-traumatic spinal cord following viral vector- or cell-mediated gene therapy. In this study we evaluated the therapeutic efficacy and changes in spinal cord after treatment with the genes encoding vascular endothelial growth factor (VEGF), glial cell-derived neurotrophic factor (GDNF), angiogenin (ANG), and neuronal cell adhesion molecule (NCAM) applied using both approaches. Therapeutic genes were used for viral vector- and cell-mediated gene therapy in two combinations: (1) VEGF+GDNF+NCAM and (2) VEGF+ANG+NCAM. For *direct gene therapy* adenoviral vectors based on serotype 5 (Ad5) were injected intrathecally and for *cell-mediated* gene delivery human umbilical cord blood mononuclear cells (UCB-MC) were simultaneously transduced with three Ad5 vectors and injected intrathecally 4 h after the SCI. The efficacy of both treatments was confirmed by improvement in behavioral (BBB) test. Molecular and cellular changes following post-traumatic recovery were evaluated with immunofluorescent staining using antibodies against the functional markers of motorneurons (Hsp27, synaptophysin, PSD95), astrocytes (GFAP, vimentin), oligodendrocytes (Olig2, NG2, Cx47) and microglial cells (Iba1). Our results suggest that both approaches with *intrathecal delivery* of therapeutic genes may support functional recovery of post-traumatic spinal cord via lowering the stress (down regulation of Hsp25) and enhancing the synaptic plasticity (up regulation of PSD95 and synaptophysin), supporting oligodendrocyte proliferation (up regulation of NG2) and myelination (up regulation of Olig2 and Cx47), modulating astrogliosis by reducing number of astrocytes (down regulation of GFAP and vimetin) and microglial cells (down regulation of Iba1).

## Introduction

To date the interest to the gene therapy is growing and motivated by results from animal research that show a potential for chronic SCI. Novel approach with recombinant genes is able to provide genes encoding neurotrophic factors, growth factors and cytokines, employing the dual or triple gene combinations, which consist of transgenes with different functions (Islamov et al., 2015). Simultaneous delivery of various therapeutic genes into post-traumatic spinal cord may not only support the viability of affected injury cells and stimulate their regeneration, but also influence different mechanisms of the SCI including inflammation, demyelination, astrogliosis, and others (Lim et al., 2010; Walthers and Seidlits, 2015). Currently, several therapeutic genes are considered to be effective for single gene therapy, i.e., encoding neurotrophic BDNF (Nakajima et al., 2007), NGF (Romero et al., 2001), GDNF (Chou et al., 2014), growth VEGF, FGF (De Laporte et al., 2011), Angiogenin (Povysheva et al., 2017), anti-apoptotic BCL-2, BCL-XL (Yukawa et al., 2002), and anti-inflammatory IL-10, IL-1RA (Zhou et al., 2009) factors, and cell adhesion NCAM and L1 molecules (Chaudhry et al., 2006; Thuret et al., 2006; Walthers and Seidlits, 2015). The search for the best single candidate or the optimal combinations of several therapeutic genes is particularly important for translation of the gene therapy strategy to clinic. In our recent study, along with other findings, we observed improvements and prolongation of lifespan in amyotrophic lateral sclerosis (ALS) mice after transplantation of the umbilical cord blood mononuclear cells (UCB-MCs) simultaneously transduced with three adenoviral vectors (Ad) encoding vascular endothelial growth factor (VEGF), glial derived neurotrophic factor (GDNF) and neural cell adhesion molecule (NCAM). Recently we also describe a positive effect of UCB-MC simultaneously transduced with three adenoviral vectors carrying VEGF, GDNF and NCAM for treatment of SCI in mini-pigs (Islamov et al., 2017) with earlier functional recovery. The role of each factor and their combination has to be further investigated. This study was specifically focused on the following factors:

### Vascular endothelial growth factor (VEGF)

Vascular endothelial growth factor (VEGF) has a known direct neuroprotective effect on rat spinal cord neurons, which could be mediated *in vitro* through VEGFR2/Flk-1 receptors (Ding et al., 2005). Importance to maintain VEGF production is dictated by its low level in the injured spinal cord (Herrera et al., 2009). It has been shown that VEGF can decrease secondary degeneration of the neurons and improves functional outcome in experimental SCI (Widenfalk et al., 2003). VEGF delivery by neural stem cells can enhance proliferation of glial progenitors, angiogenesis, and improve tissue sparing after SCI (Kim et al., 2009). Adenovirally delivered bio-engineered zinc-finger transcription factor, designed to activate expression of all isoforms of endogenous VEGF-A, resulted in an attenuation of axonal degradation, decreased level of apoptosis, a significant increase in vascularity, improvements in behavioral outcomes following SCI (Liu et al., 2010; Figley et al., 2014).

### Glial derived neurotrophic factor (GDNF)

Glial derived neurotrophic factor (GDNF) is well known factor to rescue neurons following ischemia, neurodegeneration or neurotrauma. Intraspinal injection of recombinant adenovirus carrying recombinant gene of GDNF into the injured spinal cord can preserve neuronal fibers and promoted functional recovery following SCI (Tai et al., 2003). Recently, on the rat model of SCI we demonstrated that UCB-MCs-mediated GDNF therapy can improve tissue sparing, although the number of myelinated fibers was higher compare to the number of fibers measured after direct injection of Ad-GDNF (Mukhamedshina et al., 2016b). Moreover, in this study we observed distinct molecular reactions in the different populations of glial cells in various areas of the post-traumatic rat spinal cord.

### Hypoxia-inducible factor angiogenin (ANG)

Hypoxia-inducible factor angiogenin (ANG) promotes motoneuron survival both *in vitro* and *in vivo* Kieran et al., 2008). ANG is a neuronally secreted factor that is endocytosed by astroglia and mediates neuroprotection in paracrine fashion (Skorupa et al., 2012). The role of this factor in pathophysiology of SCI is poorly understood. Major data on the role of ANG on spinal cord regeneration were received in ALS models. Thus, ALS mice injected with the Ad-VEGF+Ad-ANG combination showed a 2–3 week delay in manifestation of the disease, higher motor activity at the advanced stages of the disease, and increase in the lifespan (Ismailov et al., 2014).

### The molecule L1

The molecule L1 from the family of Ig-like cell adhesion molecule (Ig-CAM) was the most studied with respect to the problem of spinal cord injury (Jakovcevski et al., 2013). The key members of Ig-CAM family are L1 cell adhesion molecule (L1-CAM) and neural cell adhesion molecule (N-CAM), which play a critical role in surface interactions of neurons by binding to each other and to the extracellular matrix (ECM) proteins. The mechanisms of L1-CAM and N-CAM effect in regeneration are supposed to be mediated through their activation of the tyrosine kinase receptors of fibroblast growth factor (FGF), epidermal growth factor (EGF), and nerve growth factor (NGF) (Colombo and Meldolesi, 2015). Injection of adeno-associated virus (AAV) encoding the L1 cell adhesion molecule (AAV-L1) at the time of acute thoracic compression injury of adult mice promotes functional recovery and associated with ameliorated astrogliosis and axonal regeneration in the lumbar spinal cord (Lee et al., 2012). Stem cells (Chen J. et al., 2005; Cui et al., 2011) or glial cells (Lavdas et al., 2010; Xu et al., 2011) based L1 gene delivery promoted functional recovery in rodent SCI models. In this regard, NCAM is still poor investigated molecular. Astrocytic scar formation at the injury site was found to be higher in NCAM^−/−^ compared with NCAM^+/+^ mice (Saini et al., 2016) and it was suggested that transduction of UCB-MCs with Ad-NCAM can contribute to their homing and survivability after intravenous transplantation into ALS mice.

In this study we evaluated the effect of triple gene therapy on motor recovery after contusion in rat in two gene combinations (1) VEGF+GDNF+NCAM and (2) VEGF+ANG+NCAM, and also evaluated the molecular and cellular reactions in post-traumatic spinal cord after gene therapy. Earlier we have demonstrated that specific gene combinations VEGF+GDNF+NCAM improve functional outcome in amyotrophic lateral sclerosis (ALS) mouse model (Islamov et al., 2016). We also described that administration of Ad5 carrying VEGF and ANG into skeletal muscles of ALS mice decrease the manifestation of pathological signs and increased the life span (Ismailov et al., 2014). The same combination of adenoviruses was tested in a 2-year clinical trial and showed increase in life span of patients with ALS (Zavalishin et al., 2008). Based on nature of response, in VEGF+GDNF+NCAM combination, VEGF and GDNF were considered as neuroprotective factors and, in VEGF+ANG+NCAM combination, VEGF and ANG were considered as angiogenic factors. Based on these results, for present study we have selected two combinations VEGF+GDNF+NCAM and VEGF+ANG+NCAM. The effect of gene therapy in post-traumatic spinal cord may be achieved by delivery of the therapeutic genes either by viral vector-mediated or cell-mediated administration and in this study we also compared the spinal cord changes after intrathecal delivery of the triple gene combinations using adenoviral vectors vs. UCB-MCs.

## Materials and methods

### Preparation of adenoviral vectors and genetically engineered UCB-MCs

This study was reviewed and approved by an institutional review board “Kazan State Medical University Animal Care Committee” and all procedures were performed according to protocol “Work with hematopoietic stem cells. SMK-MI-02.04-07,” license FS-16-01-001421.

#### Adenoviral vectors

Adenoviral vectors carrying VEGF165, GDNF, ANG, NCAM1 and green fluorescent protein (GFP) genes were generated by the method of homologous recombination based on the human adenovirus serotype 5 (Ad5) in Gamaleya Research Institute of Epidemiology and Microbiology (Moscow, Russia) as described previously (Shcherbinin et al., 2014). For injection 2 × 10^7^ virus particles of Ad5-GFP in 20 μl of saline; 2 × 10^7^ virus particles mixture of Ad5-VEGF165, Ad5-GDNF and Ad5-NCAM1 in 20 μl of saline; and 2 × 10^7^ virus particles mixture of Ad5-VEGF165, Ad5-ANG and Ad5-NCAM1 in 20 μl of saline were prepared.

#### Mononuclear cells

Mononuclear cells from human umbilical cord blood (UCB-MCs) were isolated by standard technique of sedimentation on to a density barrier (Ficoll, 1.077g/ml) as described previously (Islamov et al., 2015) based on the license of Kazan State Medical University Stem Cell Bank. Genetically engineered UCB-MCs were obtained following transduction of the cells with Ad5-GFP at MOI 10; simultaneously with Ad5-VEGF165, Ad5-GDNF, and Ad5-NCAM1 at MOI 10; and simultaneously with Ad5-VEGF165, Ad5-ANG, and Ad5-NCAM1 at MOI 10. For injection 2 × 10^6^ UCB-MCs+Ad5-GFP in 20 μl of saline; 2 × 10^6^ UCB-MCs+Ad-VEGF165+Ad-GDNF+Ad-NCAM1 in 20 μl of saline; and 2 × 10^6^ UCB-MCs+Ad-VEGF165+Ad-ANG+Ad-NCAM1 in 20 μl of saline were prepared.

### Evaluation of transgenes expression *in vitro*

For quantitative reverse-transcription PCR (qRT-PCR) analysis UCB-MCs were harvested five days after transduction with Ad5-VEGF165, Ad5-GDNF and Ad5-NCAM1. Total RNA from genetically engineered UCB-MCs was isolated using the TRIZOL (Invitrogen). cDNA were obtained by 100 U Maxima Reverse Transcriptase (Thermo Scientific, USA). To determine the expression rate of VEGF165, GDNF and NCAM1 TaqMan method was employed. All primers and probes used in the study are listed in Table 1. Real-time PCR was performed and results were analyzed with the CFX 96 RealTime PCR System (Bio-Rad). The level of mRNA was normalized using 18S rRNA as housekeeping gene. For each sample, PCR reactions were performed in triplicate. The cDNA levels were determined using the standard curve. The standards used for the standard curve were generated using serial dilutions of plasmid DNA with corresponding cDNA inserts. Expression level of target genes in naïve (non-transduced) UCB-MC was considered as 100%.

**Table 1:** RT-PCR primers and TaqMan probes.

**Genes**	**Primer and probes**
hVEGF-Forward	TACCTCCACCATGCCAAGTG
hVEGF-Reverse	TGATTCTGCCCTCCTCCTTCT
hVEGF-TMProbe^*^	[FAM]TCCCAGGCTGCACCCATGG[BH1]
hGDNF-Forward	CGCTGAGCAGTGACTCAAAT
hGDNF-Reverse	CGATTCCGCTCTCTTCTAGG
hGDNF-Probe^*^	[FAM]TCCATGACATCATCGAACTGATCAGG[BH1]
hNCAM1-Forward	AGATGAGGGCACTTATCGCT
hNCAM1-Reverse	GATGGTAGGTGGCACATTCA
hNCAM1-Probe^*^	[FAM]CCGTGCCAGGATTCTGCCCT[BH1]
18S-TM-Forward	GCCGCTAGAGGTGAAATTCTTG
18S-TM-Reverse	CATTCTTGGCAAATGCTTTCG
18S-TM-Probe^*^	[HEX]ACCGCGCAAGACGGACCAG[BH2]

* *TaqMan probes is labeled at the 5′-end with the fluorescence FAM (HEX) and a 3′ quenching acceptor Black Hole (BH1/BH2)*.

Using enzyme-linked immunosorbent assay (ELISA) the level of soluble VEGF in the conditioned culture media after incubation of UCB-MCs transduced with Ad5-VEGF165, Ad5-GDNF, and Ad5-NCAM1 was estimated. After transduction UCB-MCs were incubated for 96 h at 37°C in a humid atmosphere, the medium was collected and centrifuged at 3,000 g for 10 min, filtered through a 0.22 μm filter. Concentration of VEGF in conditioned medium was measured with a VEGF DuoSet ELISA kit from R&D Systems (#DY293B DuoSet) according the manufacturer's protocol. UCB-MCs transduced with Ad5-GFP were examined 96 h after incubation using fluorescent microscopy to assess expression of the green fluorescent protein.

### Animals and treatments

Fifty four female Wistar rats (Pushchino Laboratory, Russia) weighing 250–300 g were used in this study. Rats were housed one per cage under standard laboratory conditions with unlimited access to food and water and a 12-h light/dark schedule. The experimental protocol was consistent with the recommendations of the Physiological Section of the Russian National Committee on Bioethics. All animal treatments, anesthesia, surgery, post-operative care, perfusion, and euthanasia at the endpoints were approved by the Kazan State Medical University Animal Care Committee (Permit Number 5, from 27 May 2014). Before surgery animals were randomly assigned to seven experimental groups (Table 2).

**Table 2:** Experimental groups.

**Group**	**Number of animals**	**Solution (20 ul) for intrathecal injection**
Saline	4	0.9% NaCl
Ad-GFP	8	2 × 10^7^ virus particles of Ad-GFP
Ad-VEGF-GDNF-NCAM	5	2 × 10^7^ virus particles of Ad-VEGF165+Ad-GDNF+Ad-NCAM1
Ad-VEGF-ANG-NCAM	4	2 × 10^7^ virus particles of Ad-VEGF165+Ad-ANG+Ad-NCAM1
UCB-MCs+Ad-GFP	6	2 × 10^6^ of UCB-MCs+Ad-GFP
UCB-MCs+Ad-VEGF-GDNF-NCAM	6	2 × 10^6^ of UCB-MCs+Ad-VEGF165+Ad-GDNF+Ad-NCAM1
UCB-MCs+Ad-VEGF-ANG-NCAM	7	2 × 10^6^ of UCB-MCs+Ad-VEGF165+Ad-ANG+Ad-NCAM1

**Figure 1:**
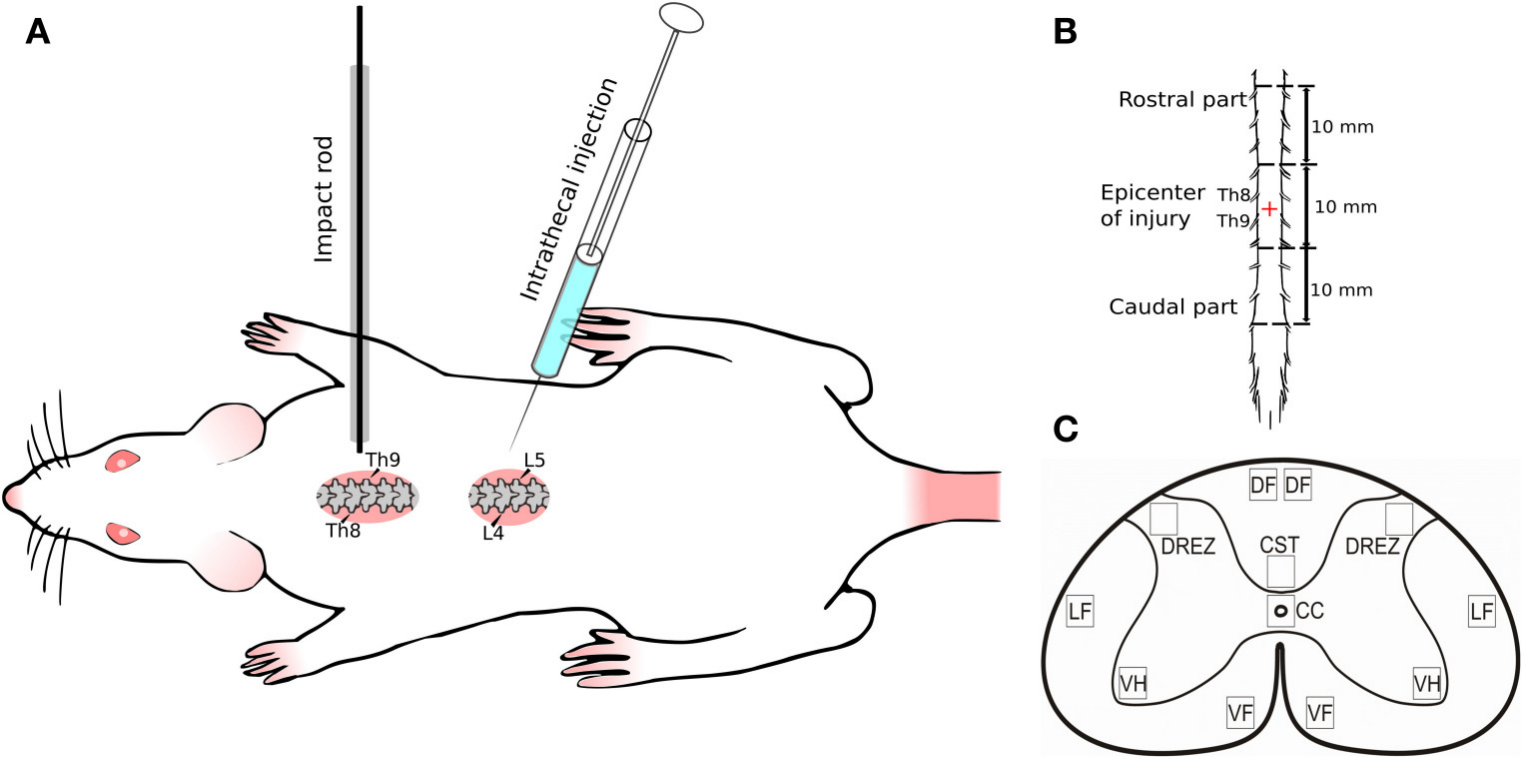
Experimental design. **(A)** Spinal cord injury and intrathecal injection; **(B)** Spinal cord fragment (30 mm) divided in three segments: rostral (10 mm) and caudal (10 mm) from the epicenter of injury (10 mm); **(C)** Spinal cord zones used for immunofluorescent staining. Seven spinal cord areas were selected: ventral horn (VH); ventral corticospinal tract (CST); dorsal root entry zone (DREZ); area of the central canal (CC); dorsal funiculi (DF); the ventral funiculi (VF); outer area of the lateral funiculi at the line passing through the central canal (LF).

All surgical procedures were performed under Zolitil 100, 3 mg/kg (Virbac Laboritaries, France) and Xyla, 4.8 mg/kg (Interchemie werken ≪De Adelaar≫ B.V≫, Netherlands) anesthesia. The model of SCI was performed in a standard way (Mukhamedshina et al., 2016b). Weight-drop injury for moderate contusion injury of spinal cord was induced using an impact rod (weight 10 g, diameter 2 mm) dropped from a height of 25 mm onto the exposed spinal cord after laminectomy at Th8–Th9 level (Figure 1A). Four hours after surgery animals were intrathecally injected between L4 and L5 vertebras either with: 20 μl of saline, 2 × 10^7^ virus particles in 20 μl of saline, or UCB-MCs (2 × 10^6^ cells in 20 μl of saline). After the surgery all animals were housed in standard conditions (21 ± 3°C, 12 h light/dark cycle) with *ad libitum* access to food and water. Rats received daily antibiotic Ceftriaxone (Sandoz, Austria) intramuscularly (50 mg/kg) and anesthetic Ketorol (Dr. Reddy's Laboratories, Ltd., Hyderabad, Andhra Pradesh, India) for 5 days post-injury (dpi). Bladder and colon were manually emptied until spontaneous voiding returned. The conditions of the rats were evaluated every day till the end point of the experiment. Daily monitoring included main criteria for euthanasia, such as body temperature (hypothermia), breathing (increased respiratory rate and effort), motor activity (immobility), and loss of ambulation (inability to access food or water), dehydration (skin pinch test), presence of large open wounds, and rapid weight loss. During this study 11 rats were euthanized under anesthesia according to the criteria for euthanasia and humane endpoints. The mortality rate of the experimental animals in early post-operative period in this study corresponded to the severity of the surgery as described previously (Zhang et al., 2016).

### Assessment of locomotor activity

All animals were assessed for open field locomotion using the Basso-Beattie-Bresnahan (BBB) score. The test for functional recovery was conducted beginning from the seventh day after surgery and was followed with daily interval and finished at 30 dpi.

### Histological assessment

For histology rats were anesthetized with chloral hydrate (80 mg/ml, 0.4 ml per 100 g; Sigma, Saint Louis, MO) and intracardially perfused with 4% paraformaldehyde (PFA, Sigma) in phosphate-buffered saline (PBS, pH 7.4) as previously described (Mukhamedshina et al., 2012, 2016a). The depth of anesthesia was assessed with pedal reflex (toe pinch) and eye blink reflex. Spinal cord fragments (30 mm) were harvested after 30 dpi and divided in three segments: epicenter of injury (10 mm), rostral (10 mm), and caudal (10 mm) from the contusion site (Figure 1B). Rostral and caudal parts were postfixed in 4% paraformaldehyde at 4°C overnight. Transverse free-floating sections (20 μm) were obtained from the site adjacent to the epicenter of injury with a cryostat (Microm HM 560, Thermo Scientific, Waltham, MA). The rostral part was chosen for studying of therapeutic gene expression to confirm the ability of adenoviral vectors and UCB-MCs to pass from injection site via epicenter to the rostral areas of the spinal cord. The caudal part (region below the contusion injury) was selected to evaluate the glial and neural cells response to gene therapy. We found that caudal part of the spinal cord had preserved morphology of white and gray matter after contusion compare to injury side with cavitations and scar formations. Thus, the cellular and molecular reaction during regeneration was evaluated primary in caudal region of the spinal cord.

For immunofluorescent analysis sections were washed in PBS with 1% Triton X-100 (Sigma) three times for 5 min, and blocked in 5% normal goat serum for 1 h at room temperature (RT). For immunofluorescent labeling sections were treated overnight at 4°C with a combination of primary antibodies (Table 3) followed by incubation in a mixture of secondary antibodies for 2 h at RT. DAPI (10 μg/ml in PBS, Sigma) was used for visualization of nuclei. Propidium Iodide solution (PI, 5 μg/ml in PBS, Sigma) was used for staining both DNA in nucleus and RNA in cytoplasm (Nissl substance in neurons) (Niu et al., 2015). Processed sections were mounted on slides, embedded in glycerol (GalenoPharm; Saint Petersburg, Russia) and observed under a LSM 510-Meta microscope (Carl Zeiss; Oberkochen, Germany).

**Table 3:** Primary and secondary antibodies used in immunofluorescent staining.

**Antibody**	**Host**	**Dilution**	**Source**
ANG	Mouse	1:150	Sigma
Cx47	Mouse	1:150	Santa Cruz
GDNF	Rabbit	1:100	Santa Cruz
GFAP	Mouse	1:200	Santa Cruz
GFAP	Rabbit	1:250	Santa Cruz
HNA (Human Nuclear Antigen)	Mouse	1:150	Millipore
HSP27	Rabbit	1:200	Abcam
NG2	Mouse	1:2000	Abcam
Olig2	Rabbit	1:150	Sigma
OSP	Rabbit	1:100	Santa Cruz
PSD95	Rabbit	1:200	Abcam
Synaptophysin	Rabbit	1:150	Abcam
VEGF	Goat	1:300	Sigma
Vimentin	Mouse	1:1200	Santa Cruz
Anti-goat IgG conjugated with Alexa 488	Donkey	1:200	Invitrogen
Anti-rabbit IgG conjugated with	Donkey	1:200	Invitrogen
Alexa 555			
Anti-mouse IgG conjugated with	Donkey	1:200	Invitrogen
Alexa 647			

The numbers of immunopositive cells (Hsp27, GFAP, Vimentin, Olig2, NG2, and Iba1) were quantified by researcher blind to examined groups of animals in the ventral horns (VH), main corticospinal tract (CST), lateral and ventral funiculies (LF and VF), area around the central canal (CC), and dorsal root entry zone (DREZ) (Figure 1C) in merged images from 10 adjacent optical slices (512 × 512 pixel resolution, observed area 0.05 mm^2^; acquisition distance, 0.5 μm). Only cells with clearly outlined nuclei were considered. Genuine co-localization was confirmed by viewing the localization of immunopositive cells on orthogonal projections of all three channels. The threshold for counting cells as co-localized was a coefficient of co-localization of at least 0.5. The level of PSD95, synaptophysin, and Cx47 expression was evaluated according the fluorescence density value corresponding markers. Digital images were obtained using a ×40 objective lens from 5 adjacent optical slices (observed area 0.09 mm^2^) with the standardized values of the pinhole, laser power and scanning speed. All images were analyzed following the same semi-automated in-house algorithm. Briefly, for each channel, the lowest intensity signals within a z-stack were removed to minimize background. Negative controls were obtained using the same protocol, but without the addition of primary or secondary antibodies.

### Statistical analyses

Immunofluorescent staining and *in vitro* molecular analysis data are presented as mean ± standard error of the mean (SEM). To determine statistical significance, we used a Student's *t*-test distribution or a one-way analysis of variance (ANOVA) with Tukey's test. A value of 0.05 was considered statistically significant. Data were analyzed using Origin 7.0 SR0 (OriginLab, Northampton, MA) software. To determine statistical significance of BBB data we used a U Wilcoxon-Mann-Whitney test using SPSS Statistics 22 (IBM, Armonk, New York, US) software, where *p* < 0.05 was considered statistically significant. Descriptive statistics for each group presented as Median (Lower Quartile; Upper Quartile). Quantitative data were presented as mean ± SEM and comparisons between groups were performed with Student's *t*-test. *p* < 0.05 was considered to indicate significant differences between groups. All analyses were performed in a “blinded” manner with respect the treatment group.

## Results

### Molecular analysis of transgene expression in UCB-MCs *in vitro*

**Figure 2:**
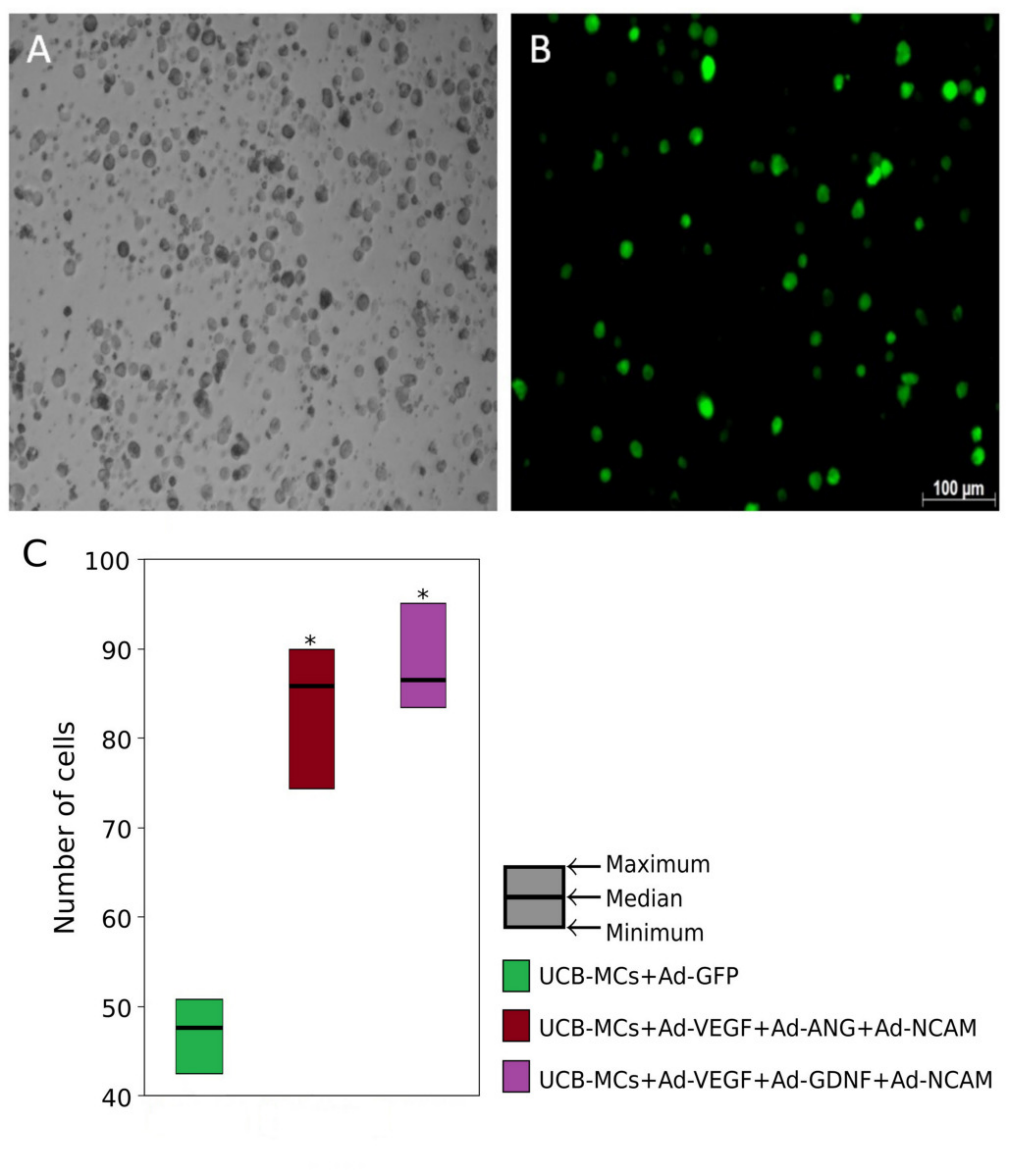
Green fluorescent protein (GFP) expression in umbilical cord blood mononuclear cells (UCB-MCs) 96 h following transduction with Ad5-GFP (multiplicity of infection = 10). **(A)** Bright field; **(B)** fluorescent field. **(C)** UCB-MCs count in post-traumatic spinal cords in the rostral direction to the injury epicenter 30 days after intrathecal injection. *Difference between groups carrying gene NCAM and the group in which UCB-MCs were transduced with reporter gene GFP, *P* < 0.05.

The mRNA level of VEGF165, GDNF, and NCAM1 in gene engineered UCB-MCs was confirmed using the RT-PCR analysis. Gene expression data were normalized to 18S ribosomal RNA level and demonstrated higher level of mRNA for VEGF165, GDNF and NCAM1 at 121.2 ± 0.47, 96.81 ± 0.64, and 118.2 ± 0.87 times correspondingly compare to non-transduced cells (*P* < 0.05). Concentrations of VEGF measured in conditioned medium of UCB-MCs+Ad-VEGF-GDNF-NCAM and UCB-MCs+Ad-GFP groups were 724.3 and 181.8 pg/ml, respectively. Fluorescent microscopy of the reporter GFP gene expression in UCB-MCs showed approximately 80% of the transduced cells (Figure 2). The GFP fluorescence in UCB-MCs was evident for a month after cells transduction with Ad5-GFP.

### Behavioral assessment

**Figure 3:**
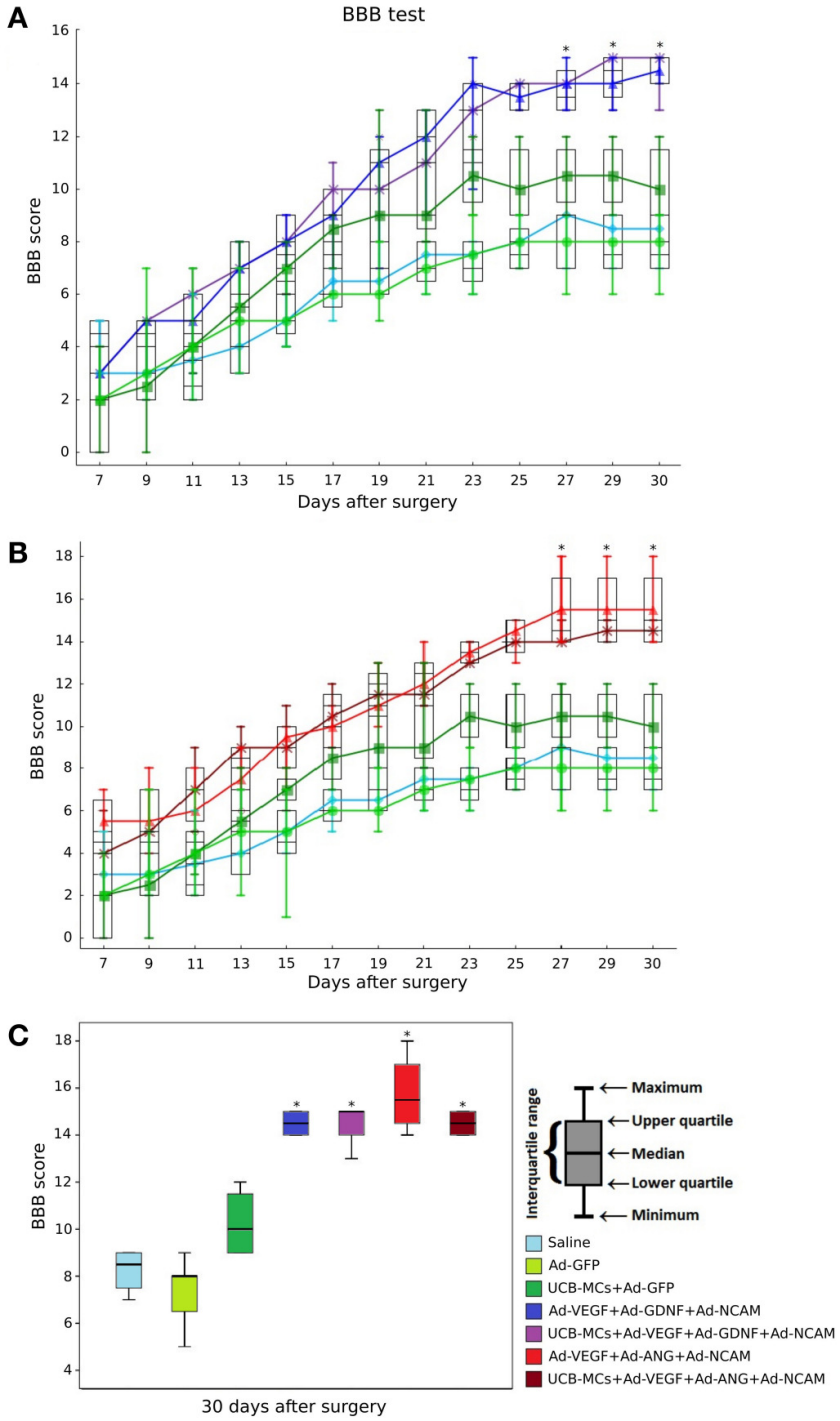
BBB performance. **(A)** Groups treated with genes encoding VEGF165, GDNF, and NCAM1; **(B)** Groups treated with genes encoding VEGF165, ANG, and NCAM1; **(C)** Comparative analysis of the BBB rate in all experimental groups at 30 day after SCI. *Difference between therapeutic and control groups, *P* < 0.05.

Recovery of the hind limbs motor activity measured with BBB score was not significantly different between experimental groups during the first 3 weeks after SCI (Figure 3). At the end of the forth week on 27 dpi statistically higher BBB score was observed in therapeutic groups (Ad-VEGF-GDNF-NCAM and UCB-MCs+Ad-VEGF-GDNF-NCAM) in comparison with control (Saline) group (*p* < 0.05). At the same time no significant difference was registered between control groups (Saline vs. Ad-GFP) and between the therapeutic groups (Ad-VEGF-GDNF-NCAM vs. UCB-MCs+Ad-VEGF-GDNF-NCAM). The rate of recovery in the cell-treated group (UCB-MCs+Ad-GFP) on 27 dpi was not significantly different from gene (VEGF, GDNF and NCAM)-treated and from control groups (Figure 3A). The results of the BBB test in therapeutic groups with genes encoding VEGF, ANG, and NCAM were similar to data presented above (Figure 3B). The greater functional improvement in therapeutic groups (Ad-VEGF-ANG-NCAM and UCB-MCs+Ad-VEGF-ANG-NCAM) in comparison with control (Saline) group was evident on 27 dpi (*p* < 0.05), however within the therapeutic groups (Ad-VEGF-ANG-NCAM vs. UCB-MCs+Ad-VEGF-ANG-NCAM) significant difference was not found. The BBB score in UCB-MCs+Ad-GFP on 27 dpi was not different from gene (VEGF, ANG, and NCAM)-treated groups (Figure 3B).

Figure 3C shows the cumulative data for all groups at 30 dpi. The BBB score was higher in all gene-treated groups in comparison with control groups (*p* < 0.05). The meanings within the gene-treated groups (Ad-VEGF-GDNF-NCAM = 14.5 [14, 15], UCB-MCs+Ad-VEGF-GDNF-NCAM = 15 [14, 15], Ad-VEGF-ANG-NCAM = 15.5 [14.75, 16.5] and UCB-MCs+Ad-VEGF-ANG-NCAM = 14.5 [14, 15]) were not different as well as the BBB score within the control groups (Saline = 8.5 [7.75, 9] and Ad-GFP = 8 [6.5, 8]). At 30 dpi the BBB score in cell-treated group (UCB-MCs+Ad-GFP = 10 [9, 11.25]) was significantly higher than in control groups and was not different from all gene-treated groups (Figure 3C).

### Immunofluorescent analysis

**Figure 4:**
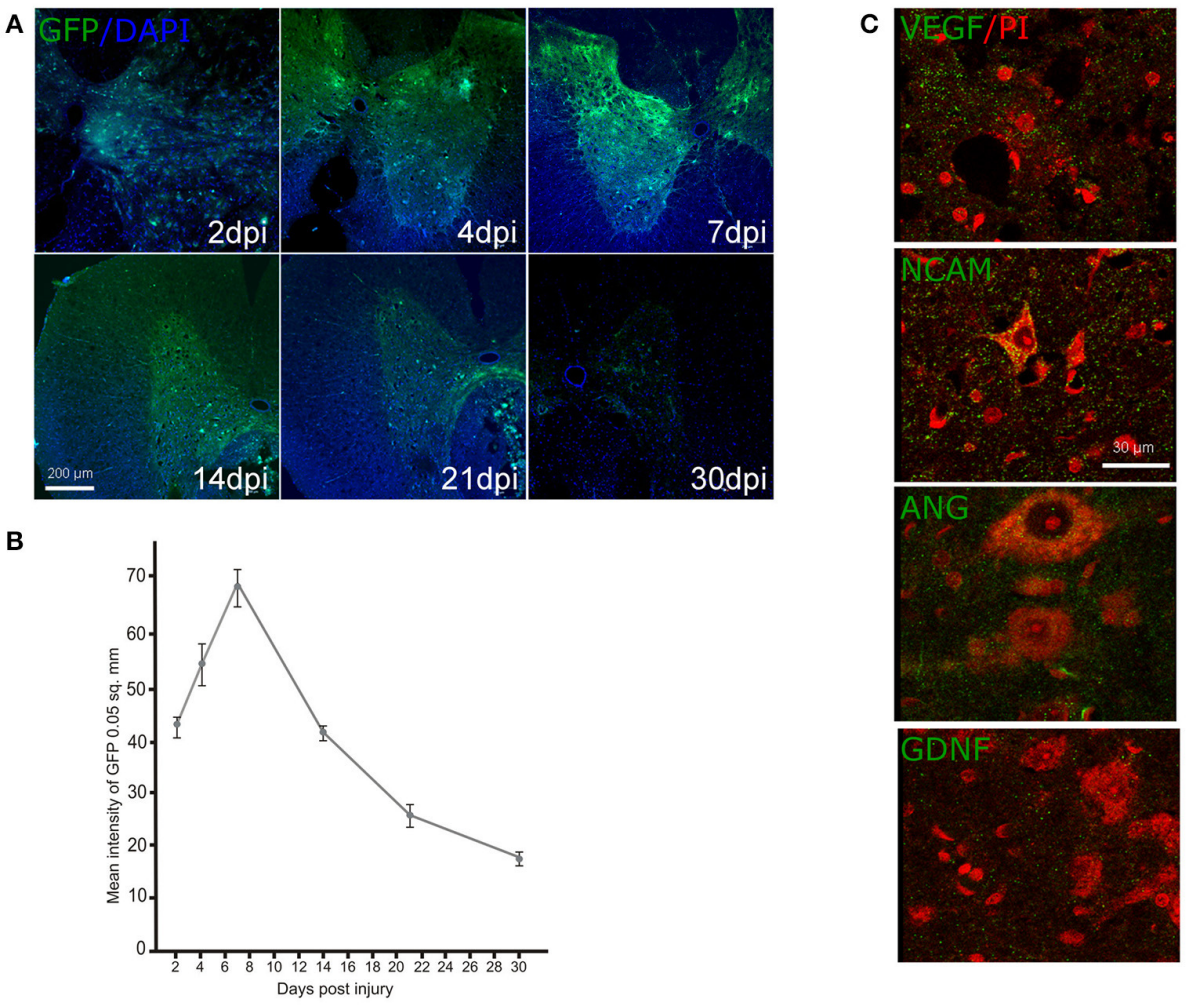
Ad-GFP, Ad-VEGF, Ad-ANG, Ad-NCAM, and Ad-GDNF transduction into the spinal cord cells. Green fluorescent protein (GFP) expression at different time points **(A)** and the mean fluorescent intensity **(B)** in the spinal cord sectioned rostrally to the site of injury after intrathecal injection of Ad-GFP. Nuclei were counterstained with DAPI (blue). Visualization of the recombinant VEGF, NCAM, ANG, and GDNF in the VH (green fluorescence) rostrally to the contusion epicenter after intrathecal injection of adenoviral vector combinations carrying corresponding genes. Neurons were counterstained with PI (red). Scale bars = 200 μm in **(A)**, 30 μm in **(C)**.

Expression of the reporter and therapeutic genes in rostral spinal cord after direct gene therapy. The expression of GFP after intrathecal injection of Ad-GFP was studied on 2, 4, 7, 14, and 21 dpi employing additional group of animals (*n* = 15) (Figures 4A,B). The mean intensity of GFP fluorescence was significantly enhanced during the first week and reached a maximum of 7 dpi. Decrease of mean intensity of GFP was observed on 14 dpi and the level of the mean intensity was continued to decrease gradually further at 21 and 30 dpi. Similar short period of expression for adenoviral vectors was described previously (Abdellatif et al., 2006). Expression of the therapeutic genes encoding VEGF, ANG, GDNF, and NCAM in the spinal cords after direct gene therapy was studied using specific antibodies against the target molecules on 30 dpi in two experimental groups: Ad-VEGF-ANG-NCAM and Ad-VEGF-GDNF-NCAM (Figure 4C). Immunofluorescent analysis reviled reconbinant molecules (VEGF, GDNF, ANG, NCAM) in post-traumatic spinal cord rostral to the injury epicenter following intrathecal injection of adenoviral vectors carrying human genes encoding VEGF, GDNF, ANG, and NCAM.

**Figure 5:**
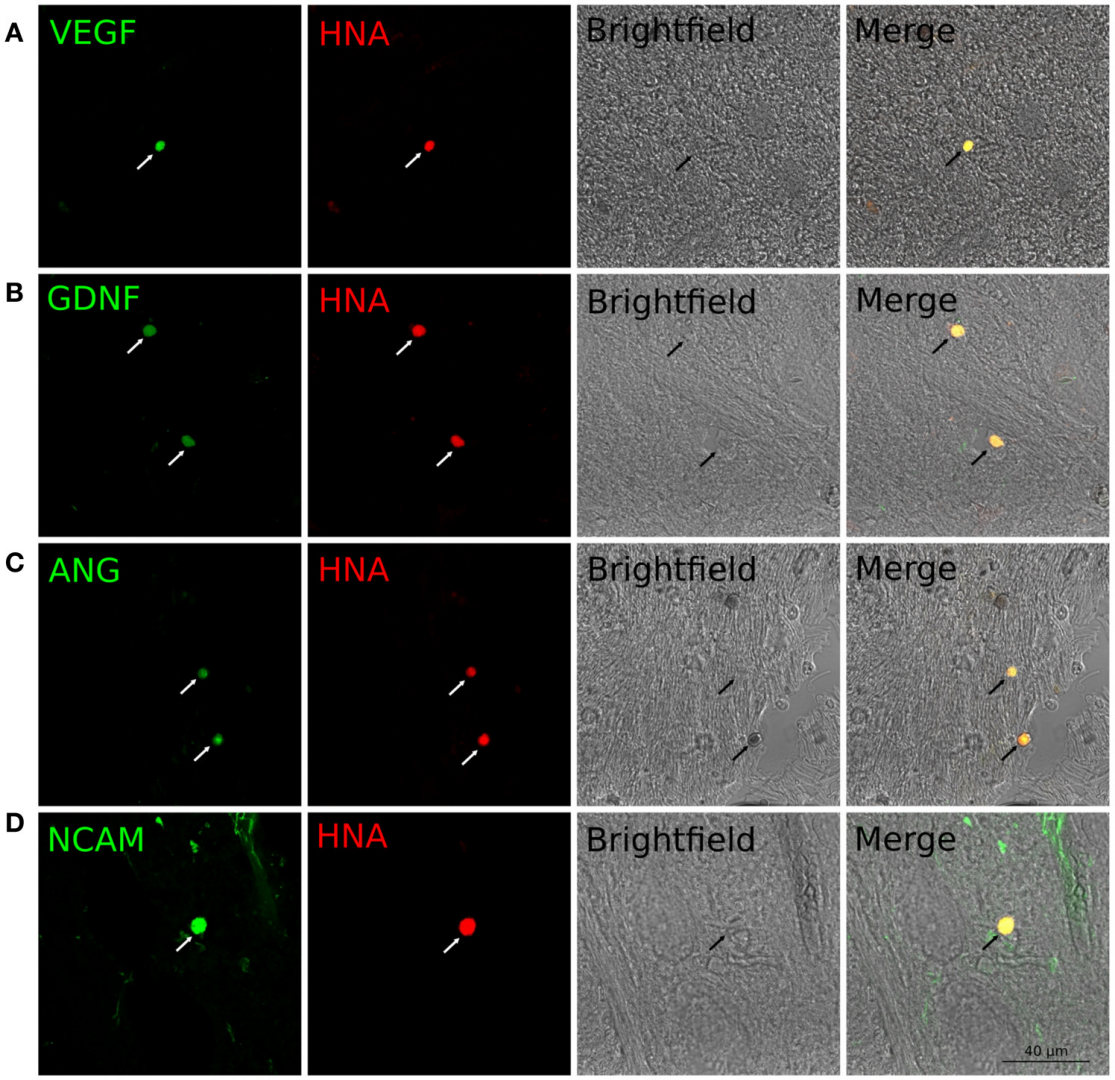
Double immunofluorescent staining of grafted UCB-MCs in rostral spinal cord at 30 day after SCI and intrathecal cell transplantation. **(A)** HNA^+^-cells (red) expressing VEGF (green). **(B)** HNA^+^-cells (red) expressing GDNF (green). **(C)** HNA^+^-cells (red) expressing ANG (green). **(D)** HNA^+^-cells (red) expressing NCAM (green). Indicated cells corresponds to the morphology of the cord blood mononuclear cells with round nuclei surrounded by a thin rim of cytoplasm.

Expression of the therapeutic genes in transplanted genetically modified UCB-MCs in the rostral spinal cord. Anti-Human Nuclear Antigen (HNA) antibodies were used for identification of human UCB-MCs in spinal cord in the rostral direction to the injury epicenter. HNA-positive cells were found in spinal cord gray and white matters of all animals with grafted UCB-MCS in the following experimental groups: (1) UCB-MCs+Ad-VEGF-ANG-NCAM, (2) UCB-MCs+Ad-VEGF-GDNF-NCAM, and (3) UCB-MCs+Ad-GFP at 30 dpi (Figure 2C). HNA-positive cells were count on 50 sections for each animal (3 rats per group). The number of UCB-MCs in UCB-MCs+Ad-VEGF-ANG-NCAM, and UCB-MCs+Ad-VEGF-GDNF-NCAM groups was not different and was 86 (75, 90) and 88 (84, 95), respectively [results are presented in the form Me (min; max). At the same time the number of UCB-MCs in these groups was significantly higher compare to the control group UCB-MCs+Ad-GFP—47 (43, 55)]. It worth to mention, that UCB-MCs after intrathecal injection at the level of L4-L5 (lower the epicenter of injury) were observed in the rostral lumbar spinal cord (above the epicenter of the injury). To detect the expression of VEGF, GDNF, ANG, and NCAM in UCB-MCs a double immunofluorescent staining with (1) HNA antibodies and (2) antibodies against one of the recombinant transgene products was used at 30 dpi. HNA-positive cells, which expressed VEGF, GDNF and NCAM, were found in the UCB-MCs+Ad-VEGF-GDNF-NCAM group and VEGF, ANG and NCAM were found in the UCB-MCs+Ad-VEGF-ANG-NCAM group. These results support the fact that genetically modified UCB-MCs are able to synthesize therapeutic molecules in spinal cord tissue up to 30 days after intrathecal transplantation (Figure 5).

### Expression of the neural and glial molecular markers in the caudal spinal cord

#### HSP27

**Figure 6:**
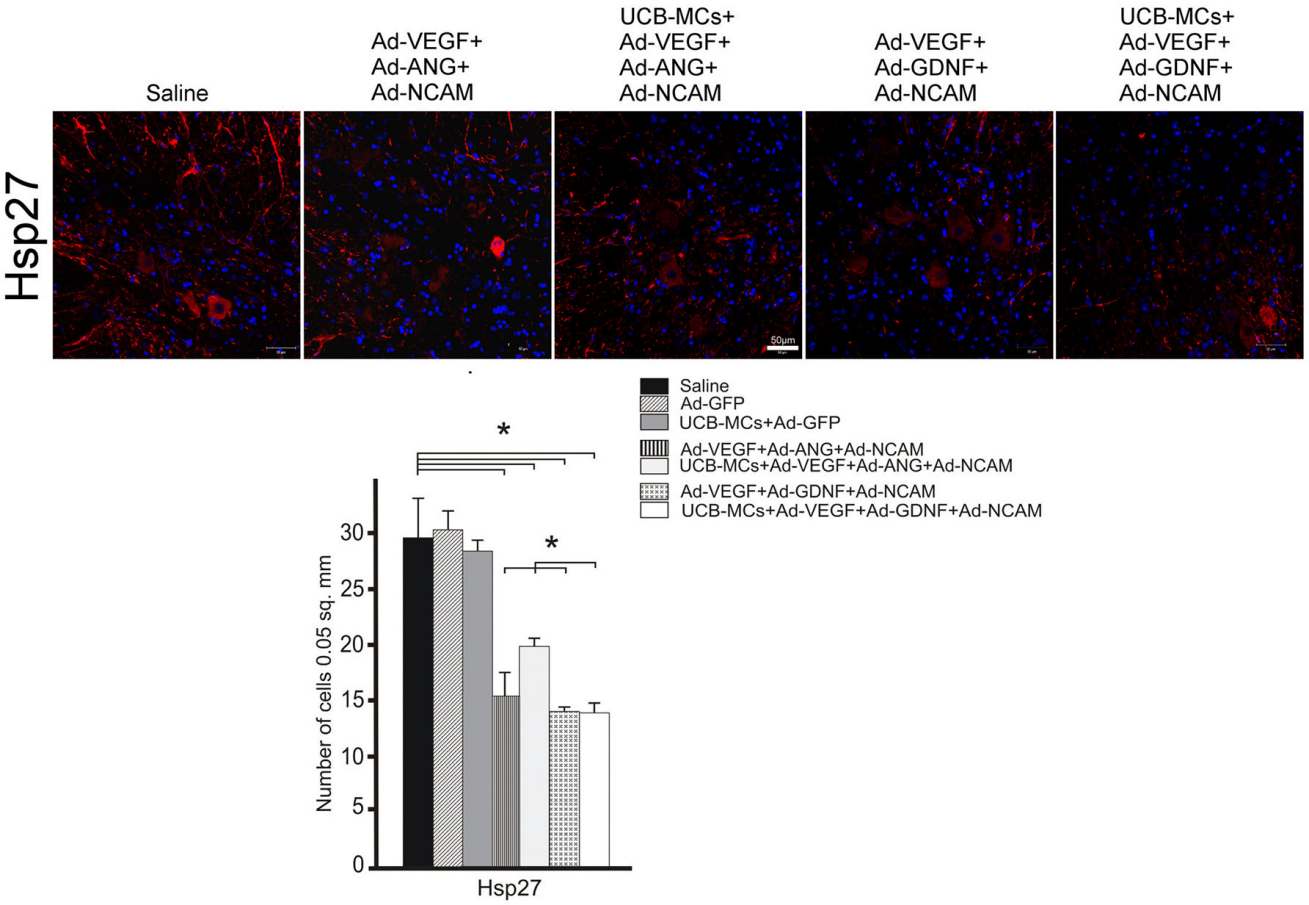
Hsp27 expression. **Upper panel:** Immunofluorescent staining of the caudal spinal cord cross sections with Abs against Hsp27 (red) caudally to the site of injury in control (Saline) and therapeutic groups. Nuclei (blue) were counterstained with DAPI. Scale bar = 50 μm. **Lower panel:** Histogram of the statistical analysis of Hsp27-positive cells number in VH zone. Data are presented as mean SEM, *p < 0.05 (one-way ANOVA).

The downregulation of the Hsp27 expression in VH was found in all therapeutic groups compare to the Saline group (29.56 ± 3.47) (Figure 6). This effect was most prominent in the UCB-MCs+Ad-VEGF-GDNF-NCAM (13.67 ± 0.85) and Ad-VEGF-GDNF-NCAM (13.84 ± 0.39) groups. The reduction of the Hsp27 expression in UCB-MCs+Ad-GFP group was not confirmed (Figure 6).

#### PSD95

**Figure 7:**
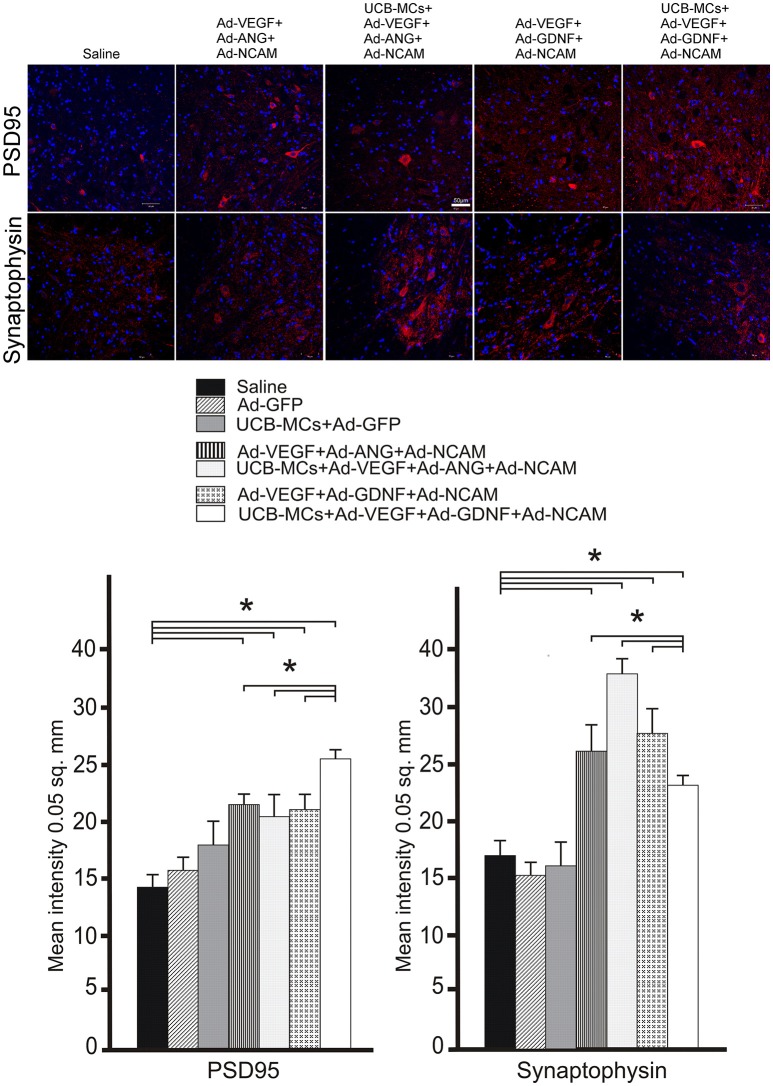
Recovery of synaptic proteins expression in the caudal spinal cord. **Upper panel:** The PSD95 and synaptophysin (red) detected with specific Abs in the VH zone caudally to the site of spinal cord injury in control (Saline) and therapeutic groups. Nuclei (blue) were counterstained with DAPI. Scale bar = 50 μm. **Lower panel:** Comparison of mean density level between different experimental groups for PSD95 and synaptophysin. Data are presented as mean SEM, *p < 0.05 (one-way ANOVA).

The level of post-synaptic marker (PSD95) expression was higher in VH in all gene-treated groups at 30 dpi compare to control groups (Figure 7). The highest level of mean density of PSD95 was in the UCB MCs+Ad-VEGF-GDNF-NCAM group (26.48 ± 2.33) in comparison with Saline group (14.27 ± 2.74). Expression of PSD95 in cell-treated group (UCB-MCs+Ad-GFP [17.11 ± 3.48]) did not differ from control groups (Figure 7).

#### Synaptophysin

Expression of synaptophysin (pre-synaptic marker) in the VH was higher in all gene-treated groups compare to control groups (Figure 7). The highest level of mean density of synaptophysin was found in UCB-MCs+Ad-VEGF-ANG-NCAM group (36.12 ± 3.44) when compared with Saline (15.32 ± 2.18) group. In the UCB-MCs+Ad-GFP group the synaptophysin expression level (15.98 ± 1.14) was not significantly different from control groups (Figure 7).

#### GFAP and vimentin

**Figure 8:**
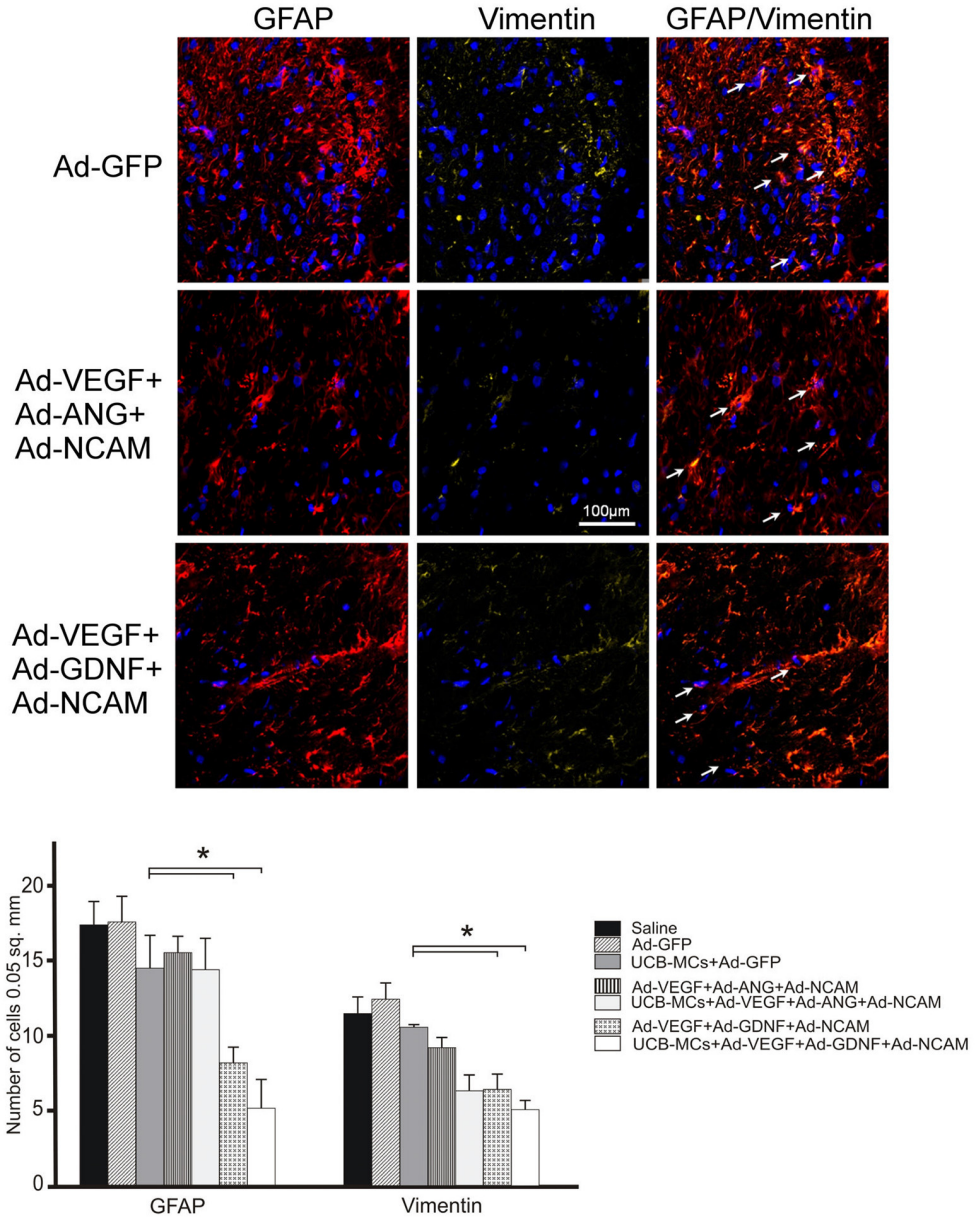
Triple gene therapy reduces reactive astrogliosis in the post-traumatic caudal spinal cord. **Upper panel:** GFAP (red) and Vimentin (yellow)-immmunoreactivity in the DREZ caudally to the SCI epicenter in Ad-GFP-, Ad-VEGF-ANG-NCAM-, and Ad-VEGF-GDNF-NCAM-treated rats. The white arrows show the co-localization of GFAP and Vimentin in astrocytes. Nuclei were counterstained with DAPI. Scale bar = 100 μm. **Lower panel:** Comparison between different groups for GFAP- and Vimentin-positive cells counted in DREZ. Data are presented as mean SEM, *p < 0.05.

The analysis of GFAP-, vimentin- and GFAP/vimentin-immunopositive astrocytes showed reduced number in DREZ in all gene treated groups in comparison to control and UCB-MCs+Ad-GFP groups (Figure 8). The most significant decrease in the number of GFAP-positive cells was documented in the groups with GDNF and particularly in Ad-VEGF-GDNF-NCAM (8.04 ± 1.19) and UCB-MCs+Ad-VEGF-GDNF-NCAM groups (6.18 ± 0.76) when compared to Saline group (17.25 ± 1.50) (Figure 8).

#### NG2 and Olig2

**Figure 9:**
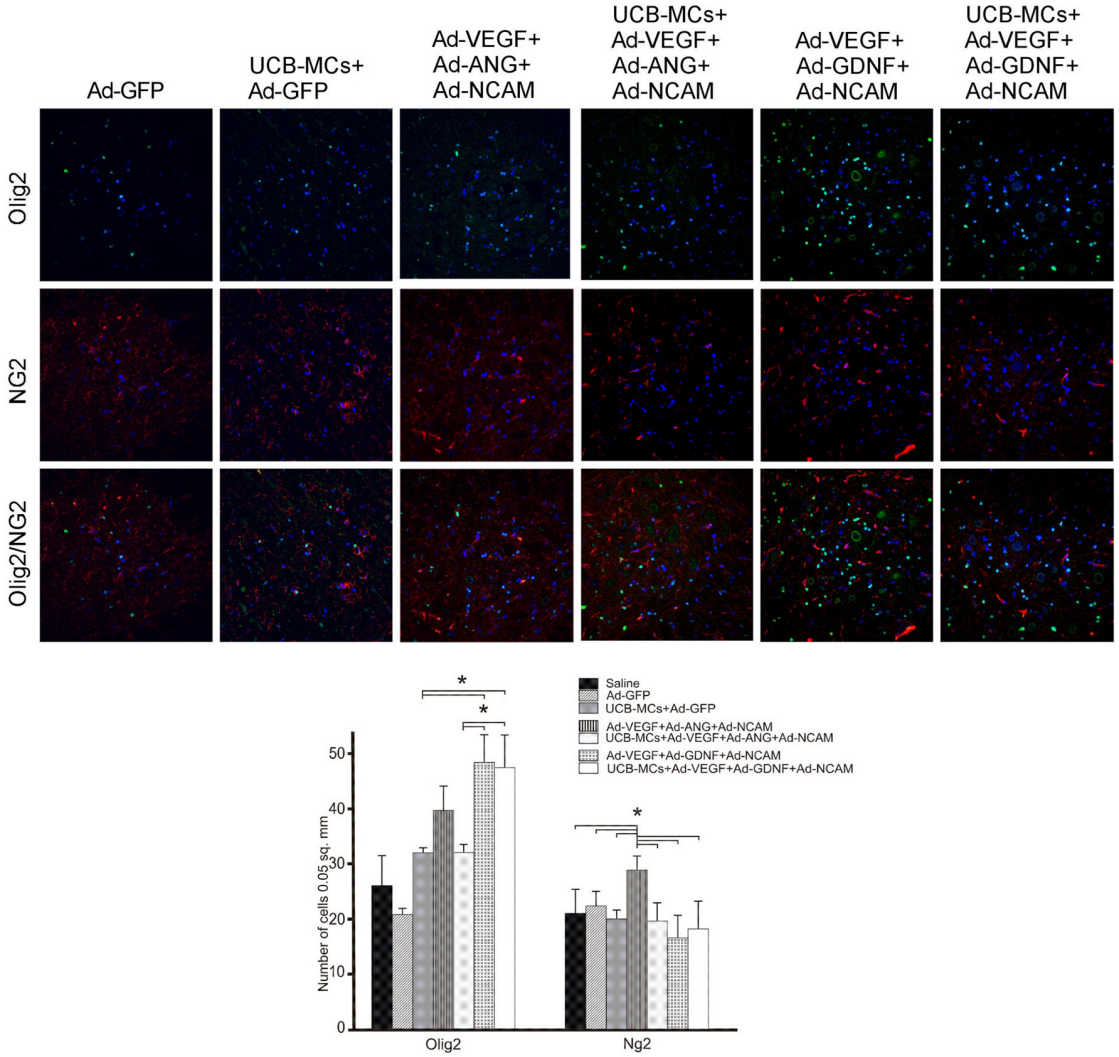
Triple gene therapy effect on the myelin-forming cells in the caudal spinal cord. **Upper panel:** Double immunofluorescent labeling showing co-localization of the oligodendrocyte precursor marker NG2 (red) with the pan-oligodendrocyte marker Olig2 (green) in VH zone of rat post-traumatic spinal cord 30 days after direct or cell-mediated gene therapy. Nuclei (blue) were counterstained with DAPI. Scale bar = 100 μm. **Lower panel:** Comparison between different experimental groups for NG2- and Olig2-positive cells counted in VH zone. Data are presented as mean SEM, *p < 0.05.

The analysis of oligodendrocyte cell line with the expression of Olig2 revealed the increased number of Olig2-immunopositive cells in DREZ and VH in groups with GDNF. Thus, in the VH the number of Olig2-positive cells in Ad-VEGF-GDNF-NCAM (48.14 ± 4.88) and UCB-MCs+Ad-VEGF-GDNF-NCAM (47.25 ± 5.91) were significantly higher in comparison with Saline (26.33 ± 5.35) and UCB-MCs+Ad-GFP (23.44 ± 1.22) groups (Figure 9). The significant increase of oligodendrocytes precursors (NG2-immunopositive cells) was found in VH in the Ad-VEGF-ANG-NCAM group (29.00 ± 2.58) when compared to other therapeutic and Saline (26.33 ± 5.35) groups. Although the number of NG2-immunopositive cells in VF was higher in all gene-treated when compared with Saline (19.80 ± 3.49) group.

The number of Olig2/NG2-immunopositive cells was decreased in gene treated groups in all studied areas of the spinal cord with exception of the CC zone. Increase of Olig2/NG2-immunopositive cells number was found in the Ad-VEGF-GDNF-NCAM (19.00 ± 2.58) and UCB-MCs+Ad-VEGF-GDNF+NCAM (16.59 ± 1.66) groups in comparison with Saline group (10.8 ± 3.00) (Figure 9).

#### Cx47

**Figure 10:**
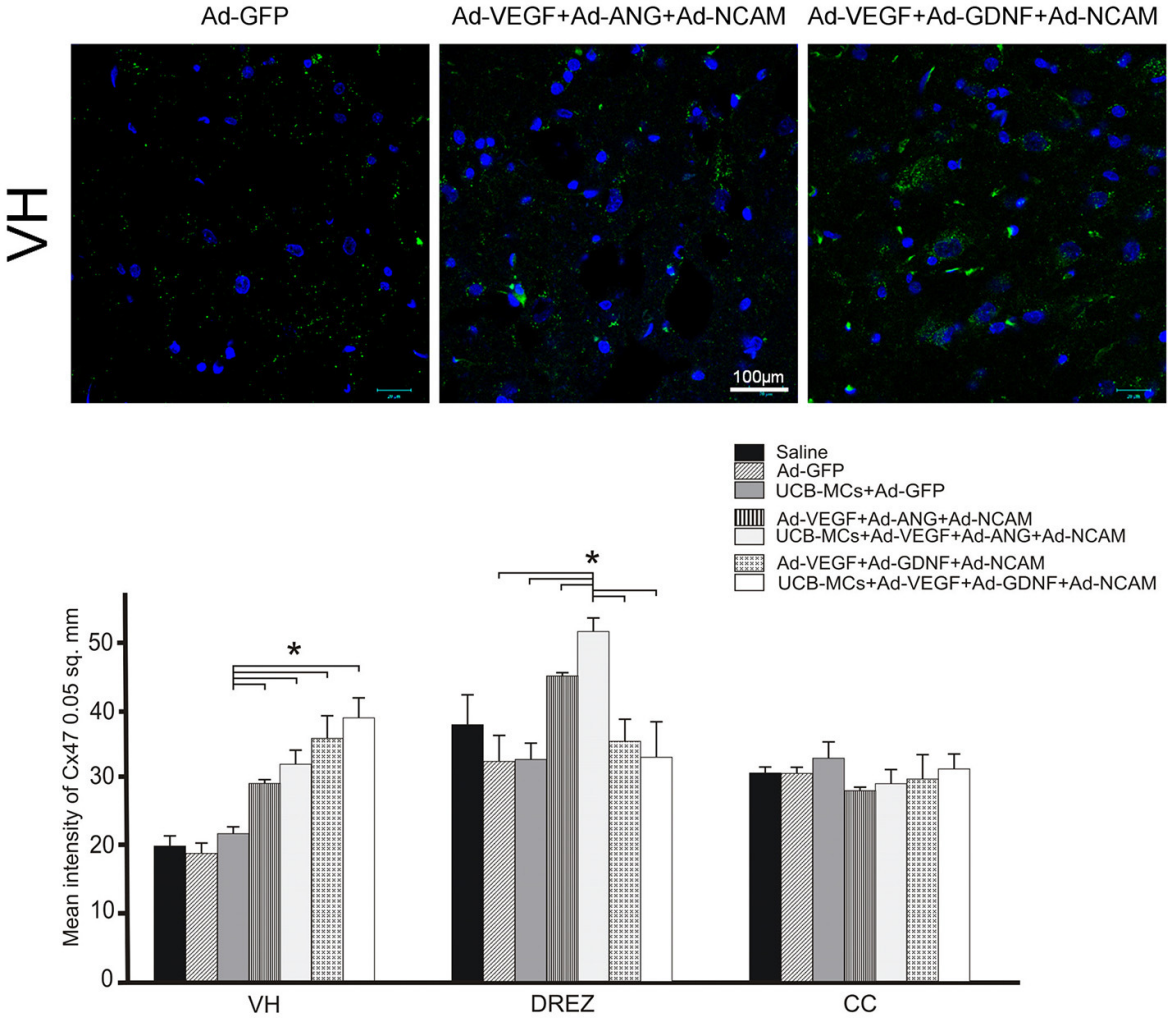
Gene therapy effect on communication between glial cells (astrocytes and oligodendrocytes) in the caudal spinal cord. **Upper panel:** Visualization of the expression of Cx47 (green). Nuclei were counterstained with DAPI. Scale bar = 100 μm. **Lower panel:** Histogram of the mean labeling intensity of Cx47 in VH, DREZ and CC of experimental animals. Data are presented as mean SEM, *p < 0.05.

The higher level of mean density of Cx47 (oligodendrocyte gap junction marker) in VH was identified in all gene treated groups when compared to the control groups (Figure 10). Interestingly, in DREZ, expression of Cx47 was significantly higher in therapeutic groups with ANG (Ad-VEGF-ANG-NCAM [45.34 ± 3.03] and UCB-MCs+Ad-VEGF-ANG-NCAM [51.80 ± 1.63]) compare to Saline (20.26 ± 1.46) group. The level of the oligodendrocyte gap junction marker expression in UCB-MCs+Ad-GFP group in DREZ (32.97 ± 2.46) was not significantly different from Saline group.

#### Iba1

**Figure 11:**
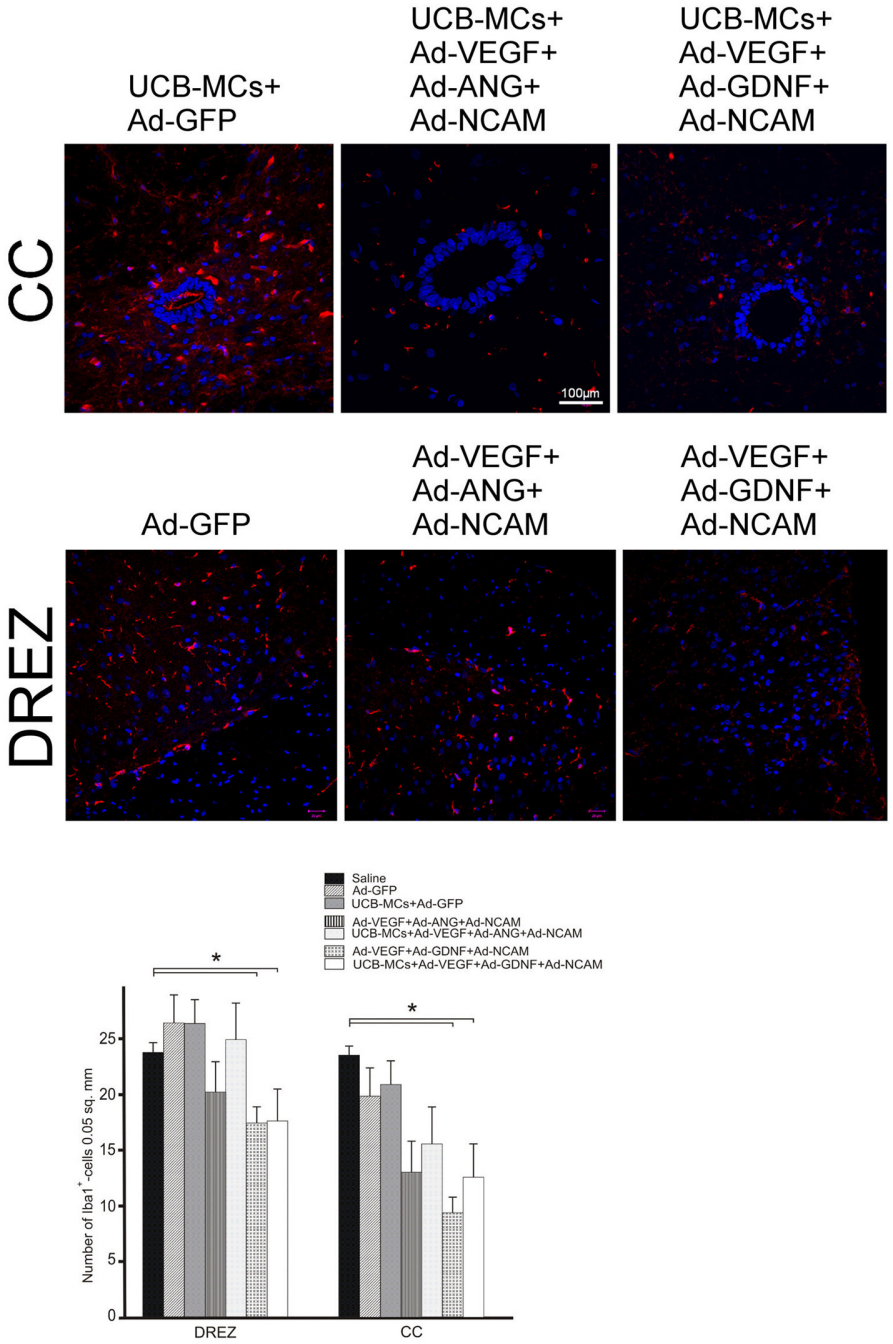
Iba1 expression in the post-traumatic caudal spinal cord. **Upper panel:** Immunofluorescent staining of spinal cord cross sections with Abs against Iba1 (red) caudally to the site of injury in CC and DREZ. Nuclei (blue) were counterstained with DAPI. Scale bar = 100 μm. **Lower panel:** Comparison between different groups for Iba1-positive cells number. Data are presented as mean SEM, *p < 0.05.

The number Iba1-immunopositive cells (microglia cells) was lower in groups with GDNF gene, in DREZ (Ad-VEGF+GDNF-NCAM [16.87 ± 2.55] vs. Saline [23.00 ± 0.95] in 1.4-fold and UCB-MCs+Ad-VEGF-GDNF-NCAM [17.05 ± 1.23] vs. Saline [23.00 ± 0.95] in 1.3-fold) and in CC (Ad-VEGF-GDNF-NCAM [9.17 ± 1.85] vs. Saline [22.67 ± 1.76] in 2.5-fold and UCB-MCs+Ad-VEGF-GDNF-NCAM [12.22 ± 1.36] vs. Saline [22.67 ± 1.76] in 1.6-fold) at 30 dpi (Figure 11). The number of microglia cells in UCB-MCs+Ad-GFP group in DREZ (25.43 ± 1.54) and CC (20.20 ± 2.92) areas did not differ from Saline group (Figure 11).

## Discussion

Many current pre-clinical studies utilize two main approaches for CNS therapeutic gene delivery—viral- and cell-mediated. Most commonly used invasive technique with direct injection of viral vectors carrying recombinant genes lead to the local transgene expression and as a result targeting the cells in the injection site. Another approach with subdural gene delivery has been considered to be less invasive and more attractive. With this approach, cerebrospinal fluid (CSF) flow helps with wider dissemination of the viral vectors throughout the CNS and also facilitates the widespread diffusion of the produced recombinant molecules. Less invasive subdural injection is more feasible for clinical application and numerous reports are addressing intraspinal transplantation (at site of lesion) of the gene-cell constructs. MSC, neural progenitor cells, Schwann cells or fibroblast were also used as carriers of genes encoding neurotrophic factors (BDNF, GDNF, CNTF, NT3 and other) (Walthers and Seidlits, 2015). Neural progenitor cells (Lepore et al., 2005) and MSC (Bakshi et al., 2004) derived from bone marrow were delivered into the injured spinal cord by lumbar puncture. Of note, intraspinal transplantation of naïve UCB-MNs (Zhu et al., 2016) and intrathecal administration of different populations of UCB cells (Ichim et al., 2010; Liu et al., 2013; Yao et al., 2013) have been already employed in clinical trials for treatment of SCI.

### Functional recovery

Previously we reported positive results with a novel therapeutic strategy for SCI treatment in mini pigs with SCI where the intrathecal administration of gene modified UCB-MNs improved nervous tissue sparing and locomotor functional recovery (Islamov et al., 2017). In the present investigation we applied the same strategy using rat model of SCI. The aim of this study was to evaluate the molecular and cellular changes in post-traumatic spinal cord in response to intrathecal administration of triple gene combinations using adenoviral vectors or UCB-MNs as carriers of the therapeutic genes. Similar to our study on mini pig, we found functional improvement in rats with SCI after intrathecal administration of adenoviral vectors or genetically engineered UCB-MCs with two combinations of therapeutic genes: (1) VEGF+GDNF+NCAM and (2) VEGF+ANG+NCAM that was demonstrated based on Basso-Beattie-Bresnahan (BBB) score. The pattern of motor recovery in gene-treated groups was consistent with our previous studies showing improvement after intraspinal injection of Ad5-VEGF+Ad5-ANG vectors on BBB score (Povysheva et al., 2017). Thus, intrathecal delivery of the therapeutic genes was found to be as effective as intraspinal approach of gene administration.

### Molecular and cellular reaction

The pathologic events, commonly observed in spinal cord after contusion injury include the death of cells (neurons, glial cells, and their precursors), followed by secondary destructive processes, such as neuritis demyelination and glial scarring, induced by reactive astrocytes and microglial cells. In this study we have demonstrate various cellular and molecular events that reflect the positive effect of triple gene therapy after SCI. In this study evaluation of the molecular and cellular reactions in post-traumatic spinal cord to gene therapy was performed based on the immunofluorescent method. For this specific neuronal (Hsp27, PSD95 and synaptophysin), oligodendrocytic (Olig2, NG2, and Cx47), astrocytic (GFAP, vimentin), and microglial (Iba1) markers were used. The level of the target molecules expression was estimated as the therapeutic genes effect on the spinal cord tissue regeneration.

#### Heat shock protein 27

Heat shock protein 27 belongs to the family of molecular chaperons with the specific function to increase the survivability of cells in condition of stress. In the CNS induction of Hsp27 occurs within defined spatial and temporal parameters in response to various pathological conditions (Franklin et al., 2005). Increased expression of Hsp27 was described in lumbar motoneurons after axotomy (Islamov et al., 2003) and cortical neurons after ischemia (van der Weerd et al., 2010). The increased level of Hsp27 is related to the severity of SCI, in contrast the decrease of Hsp27 expression in all therapeutic groups in this study suggests the positive effect of the gene therapy on survivability of VH neurons. These results are consistent with the expression of pre- and post-synaptic proteins. Thus, the level of *PSD95* and *synaptophysin* (excluding UCB-MCs+Ad-GFP group) in VH was higher than in control groups. Base on these results, motoneurons in gene treated rats may have lower stress and higher recovery of synaptic function.

#### Astrogliosis

Astrogliosis is one of the known SCI pathogenic factors. Astrocytic markers (GFAP, vimentin) in DREZ in this study showed significantly decreased expression at the site of contusion in gene treated rats. Both molecules are associated with intermediate filaments, but GFAP is considered to be a specific marker for mature cells and for vimentin—for differentiating astrocytes (Schnitzer et al., 1981). Thus, the gene therapy may potentially control proliferation and differentiation of astrocytes and prevent development of astrogliosis. The possible impact in the reduction of astrogliosis can be related with microlia. The induction of astrogliosis by activated microglia is well-known fact (Röhl et al., 2007; Tilleux et al., 2007). In our study in DREZ of gene treated rats we observed decrease in number of Iba1-immunopositive cells, which could be also considered as an inhibiting factor for astrogliosis. Accordingly, via reduction in DREZ of astrocytes and microglia, gene therapy may prevent the astrogliosis at the site of contusion.

To evaluate the myelination we used oligodendrocytic markers for mature oligodendrocytes (Olig2 and Cx47) and for their precursors (NG2). Our data demonstrates that in gene treated rats there was higher number of Olig2-immunopositive cells in DREZ and VH. In the same zones of the spinal cord we observed the elevated level of the oligodendrocyte gap junction marker (Cx47) expression. All gene treated rats had increased number of NG2-immunopositive cells in VF. The highest level of Olig2/NG2-immunopositive cells was also found in CC. The results show that gene therapy activates proliferation of oligodendrocytes precursors in CC and supports myelination in DREZ, VH, and VF.

### Comparison of gene and gene-cell constructs

The comparative analysis was performed based on the (1) presence of GDNF or ANG in triple gene combinations and (2) the way the therapeutic genes were delivery—viral vector- or cell-mediated. The presence of GDNF gene in studied gene constructs had a prominent effect on expression of Hsp27, synaptophysin, PSD95, Olig2, Cx47, vimentin, GFAP, and Iba1. Gene combination containing ANG gene revealed a superior effect on expression of synaptophysin, NG2 and Cx47. Pair-wise comparison of viral vector- and cell-mediated treatments with the same gene combinations (Ad-VEGF-GDNF-NCAM vs. UCB-MCs+Ad-VEGF-GDNF-NCAM and Ad-VEGF-ANG-NCAM vs. UCB-MCs+Ad-VEGF-ANG-NCAM) revealed some advantages and disadvantages on the expression of different neuronal and glial markers. The response of the particular markers to gene therapy (viral- or cell-mediated) was uneven in different areas of the spinal cord potentially because of the localization in relation to the contusion site, target cells type ratio, and features of intercellular communication. Meanwhile, the UCB-MCs+Ad-VEGF-GDNF-NCAM construct in general demonstrated the superior effect in VH area on stress (Hsp27), synaptic plasticity (PSD95), myelinization (Olig2, Cx42), and in DREZ area on astrogliosis (GFAP, vimentin, Iba1).

In our previous study we have shown that at the 30th post-operative day, equal positive locomotor recovery was observed after both intraspinal injection of Ad-GDNF and UCB-MCs+Ad-GDNF. However, after UCB-MCs-mediated GDNF therapy, the area of preserved tissue and the number of spared myelinated fibers were higher compare to those measured after direct GDNF gene therapy (Mukhamedshina et al., 2016b). The present results are also in line with data demonstrating tissue sparing after cell-mediated gene therapy and can be explained not only by the positive influence of delivered transgenes, but also by the effect of the UCB-MCs. The rationality for using of UCB-MCs in cell therapy for neurodegenerative disorders is highly supportive by experimental models (Zhu et al., 2016). The efficacy of UCB-MCs is based on their homing toward the neurodegeneration loci, production of growth and neuroprotective factors, ability to give rise to endothelial and microglial cells (Islamov et al., 2015). The employment of UCB-MCs as carriers for genes encoding neurotrophic factors may significantly enhance their therapeutic efficacy (Ikeda et al., 2004; Chen H. K. et al., 2005). The gene encoding neuronal cell adhesion molecule NCAM for gene engineering of UCB-MCs we used to improve migration potential and increase viability of the transplanted UCB-MCs in the spinal cord of experimental rats. Here we demonstrated that after the intrathecal injection the number of UCB-MCs expressing recombinant NCAM in post-traumatic spinal cord was higher in comparison with UCB-MCs carrying reporter gene GFP.

## Conclusion

Numerous studies demonstrated the positive effect of direct and cell-mediated gene therapy on improvement of motor performance and morphological characteristics in animal models of SCI, however, the molecular and cellular changes leading to the spinal cord recovery after gene therapy still need future investigations. In the present work we demonstrate correlation between recovery of motor functions and specific molecular and cellular changes in post-traumatic spinal cord following viral vector- and cell-mediated triple gene therapy. Our results suggest that both vector- and cell-mediated therapies with intrathecal delivery of therapeutic genes may facilitate functional recovery via down regulation of heat shock proteins (Hsp27) and up regulation of synaptic proteins (PSD95 and synaptophysin) in spinal cord neurons. Other factors they may contribute to recovery may include increasing number of oligodendrocyte (up regulation of NG2, Olig2, and Cx47), reducing number of astrocytes (down regulation of GFAP and vimetin), and microglial cells (down regulation of Iba1).

## Author contributions

RI, YC, and IL designed model framework and wrote the manuscript. AI, TP, FB, MS, FF, RG, BN, DL, and IS conducted experiments, data collection and analyzed data and contributed to manuscript writing.

### Conflict of interest statement

The authors declare that the research was conducted in the absence of any commercial or financial relationships that could be construed as a potential conflict of interest.
